# Machine learning-based model for predicting inpatient mortality in adults with traumatic brain injury: a systematic review and meta-analysis

**DOI:** 10.3389/fnins.2023.1285904

**Published:** 2023-12-14

**Authors:** Zhe Wu, Jinqing Lai, Qiaomei Huang, Long Lin, Shu Lin, Xiangrong Chen, Yinqiong Huang

**Affiliations:** ^1^Department of Neuronal Surgery, The Second Affiliated Hospital of Fujian Medical University, Quanzhou, Fujian, China; ^2^Department of Anesthesiology, The Second Affiliated Hospital of Fujian Medical University, Quanzhou, China; ^3^Department of Neurosurgery, Fuzong Clinical Medical College, Fuzhou, Fujian, China; ^4^Centre of Neurological and Metabolic Research, The Second Affiliated Hospital of Fujian Medical University, Quanzhou, Fujian, China; ^5^Department of Endocrinology, The Second Affiliated Hospital of Fujian Medical University, Quanzhou, Fujian, China

**Keywords:** traumatic brain injury, machine learning, mortality predictor, meta-analysis, inpatient mortality

## Abstract

**Background and objective:**

Predicting mortality from traumatic brain injury facilitates early data-driven treatment decisions. Machine learning has predicted mortality from traumatic brain injury in a growing number of studies, and the aim of this study was to conduct a meta-analysis of machine learning models in predicting mortality from traumatic brain injury.

**Methods:**

This systematic review and meta-analysis included searches of PubMed, Web of Science and Embase from inception to June 2023, supplemented by manual searches of study references and review articles. Data were analyzed using Stata 16.0 software. This study is registered with PROSPERO (CRD2023440875).

**Results:**

A total of 14 studies were included. The studies showed significant differences in the overall sample, model type and model validation. Predictive models performed well with a pooled AUC of 0.90 (95% CI: 0.87 to 0.92).

**Conclusion:**

Overall, this study highlights the excellent predictive capabilities of machine learning models in determining mortality following traumatic brain injury. However, it is important to note that the optimal machine learning modeling approach has not yet been identified.

**Systematic review registration:**

https://www.crd.york.ac.uk/PROSPERO/display_record.php?RecordID=440875, identifier CRD2023440875.

## 1 Introduction

Traumatic brain injury (TBI) has a high rate of disability and mortality and is one of the leading causes of death worldwide (Capizzi et al., 2020). Predicting mortality from TBI is essential for making informed clinical decisions and providing guidance to patients’ families. Traditional statistical methods have been commonly used for this purpose. However, in recent years, there has been a surge in research using machine learning (ML) to predict mortality from TBI.

ML algorithms can autonomously learn from data, generate patterns, and use these patterns to predict unknown outcomes. As a result, various ML-based models for predicting mortality in TBI have emerged (Moyer et al., 2022; Bischof and Cross, 2023; Wu et al., 2023). However, the predictive performance of these models varies across multiple studies due to factors such as the inclusion of different sample data and the use of different types of ML models. In this context, we conducted a meta-analysis to evaluate the effectiveness of ML in predicting TBI mortality and better characterize the overall performance of these models.

## 2 Methods

Our study was registered with PROSPERO (CRD2023440875) and was conducted in accordance with the guidelines provided by the Preferred Reporting Items for Systematic Reviews and Meta-Analyses (PRISMA) and PRISMA-2020 (Page et al., 2021). The review was based on a systematic search and predefined inclusion and exclusion criteria. Meta-analyses were carried out according to a predetermined analysis plan.

### 2.1 Search strategy

Systematic literature searches using PubMed, Web of Science, and Embase followed PRISMA guidelines (from inception to May 2023). Our search strategy uses medical topic headlines and natural language text terms, and search formulas are provided in the Supplementary Table.

### 2.2 Selection process

This meta-analysis excluded non-English studies and non-original studies. Studies involving pediatric populations, animals, all enrolled patients who received a specific treatment, or all enrolled patients who developed a specific TBI complication were also excluded. Additionally, studies that did not use machine learning for prediction were excluded; these studies focused primarily on assessing risk factors rather than predicting prognosis and lacked sufficient data to infer the performance of the machine learning models. In terms of outcomes, studies that predicted mortality for more than 6 months were excluded. Two authors (WZ and LJQ) independently screened each search record and removed duplicate studies using Endnote X9. Full-text assessment was performed if it was challenging to determine eligibility based on title and abstract alone.

### 2.3 Data extraction

Evaluation of the model’s performance focused on its ability to accurately discriminate between in-hospital mortality or mortality within 6 months of TBI. Two authors (WZ and LJQ) independently extracted data using the Checklist for critical Appraisal and data extraction for systematic Reviews of prediction Modeling Studies (CHARMS) checklist. In cases of disagreement, a third party assisted in the adjudication or facilitated the process of reaching consensus.

### 2.4 Risk of bias assessment

The quality and applicability of the included studies were assessed using the Predictive Model Risk of Bias Assessment Tool (PROBAST) (Wolff et al., 2019). Two review authors (WZ and LJQ) independently evaluated the studies based on four domains: participants, predictors, outcomes, and analysis.

### 2.5 Statistical analysis

Data were synthesized and analyzed using Stata 14.0 (Stata Corporation, College Station, TX, USA) software. Sensitivity and specificity were measured using the corresponding 95% confidence intervals (CIs). Additionally, a summary ROC (sROC) curve with a 95% CI was generated using a hierarchical summary receiver operating characteristic (HSROC) model to assess the collective discriminatory performance of published post-TBI mortality prediction models (Reitsma et al., 2005). A *p*-value of < 0.05 was considered statistically significant. To quantify statistical heterogeneity between studies, *I*^2^ and Cochran Q statistics were utilized. Furthermore, meta-regression and subgroup analyses were carried out to explore potential sources of heterogeneity among studies (Ioannidis, 2008).

## 3 Results

**FIGURE 1:**
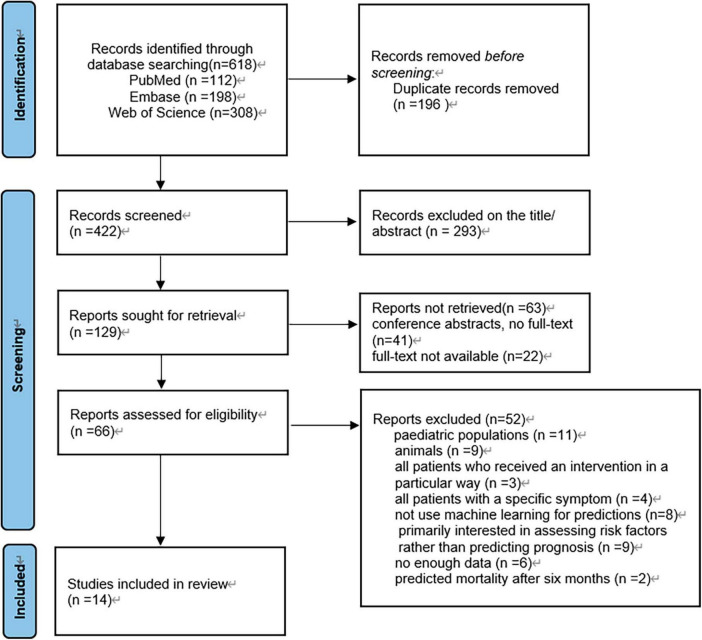
Article selection flow diagram. PRISMA (preferred reporting items for systematic reviews and meta-analyses) flow diagram for study selection.

The search strategy yielded a total of 618 articles from three databases: PubMed, Embase, and Web of Science (Figure 1). Initially, 196 duplicate articles were removed. Based on the evaluation of titles and abstracts, 293 irrelevant studies were excluded. Subsequently, 63 conference articles and articles lacking full text were excluded. Finally, 52 studies were excluded following a full-text assessment. Ultimately, 14 studies met the eligibility criteria and were included in this review.

### 3.1 Description of included studies

#### 3.1.1 Study characteristics

The earliest included studies were published in 1997, while the majority of studies were published between 2020 and 2023. These studies were conducted in different countries on different continents, with nine studies from Asia (Rau et al., 2018; Abujaber et al., 2020; Matsuo et al., 2020, 2023; Lee et al., 2022; Tu et al., 2022; Wang et al., 2022; Song et al., 2023; Wu et al., 2023), four from North America (Lang et al., 1997; Pease et al., 2022; Satyadev et al., 2022; Warman et al., 2022), two from Europe (Güiza et al., 2013; Wu et al., 2023), and one from Africa (Warman et al., 2022). Of the included studies, eight were retrospective (Güiza et al., 2013; Abujaber et al., 2020; Matsuo et al., 2020, 2023; Lee et al., 2022; Pease et al., 2022; Satyadev et al., 2022; Song et al., 2023), three were prospective (Lang et al., 1997; Warman et al., 2022; Wu et al., 2023), and three did not provide a clear description (Rau et al., 2018; Satyadev et al., 2022; Wang et al., 2022). Six studies did not specify criteria for inclusion or exclusion of patient data (Güiza et al., 2013; Rau et al., 2018; Satyadev et al., 2022; Warman et al., 2022; Wu et al., 2023), all of which included more than 200 cases. Eight of these studies exceeded 1,000 cases (Lang et al., 1997; Rau et al., 2018; Abujaber et al., 2020; Satyadev et al., 2022; Tu et al., 2022; Warman et al., 2022; Matsuo et al., 2023; Song et al., 2023; Wu et al., 2023), nine focused on in-hospital mortality outcomes (Rau et al., 2018; Abujaber et al., 2020; Matsuo et al., 2020, 2023; Tu et al., 2022; Wang et al., 2022; Warman et al., 2022; Song et al., 2023; Wu et al., 2023), four on 6-month mortality outcomes (Lang et al., 1997; Güiza et al., 2013; Pease et al., 2022; Satyadev et al., 2022), and one on 14-day mortality outcomes (Supplementary Table 1; Lee et al., 2022).

#### 3.1.2 Types of machine learning

**FIGURE 2:**
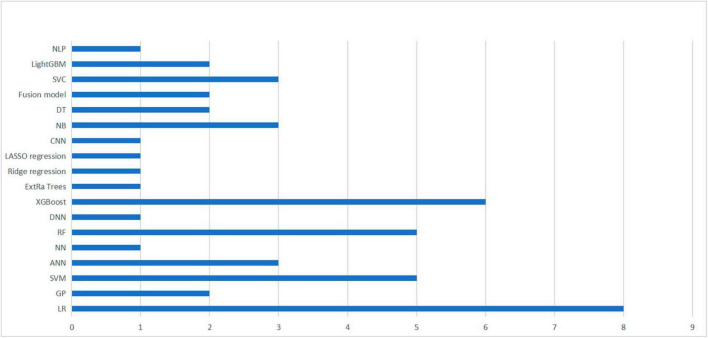
Summary of machine learning methods used in 14 studies. A summary of machine learning methods used to build TBI mortality prediction models.

In the included studies, except for Wu et al. (2023), the authors used two or more different machine learning methods to construct multiple predictive models within the same study. These models were then compared to determine the best performing machine learning algorithm. Figure 2 provides an overview of the machine learning algorithms used, with a total of 18 algorithms from 14 studies included studies.

Nonetheless, logistic regression remained the most commonly utilized method, performing best in two studies (Lang et al., 1997; Tu et al., 2022). Additionally, four studies identified XGBoost as the optimal algorithm for constructing prediction models (Wang et al., 2022; Warman et al., 2022; Matsuo et al., 2023; Wu et al., 2023), followed by SVM (Abujaber et al., 2020; Lee et al., 2022), and RF (Matsuo et al., 2020; Satyadev et al., 2022), respectively, which were considered to be the best performing models in both studies. It is worth noting that the selection of an appropriate machine learning algorithm does not completely determine the performance of the model, as it may also be influenced by the included predictors, the choice of hyperparameters and various other factors (Greener et al., 2022).

#### 3.1.3 Model performance and validation

Performance metrics, including accuracy, sensitivity, specificity, AUC, and F1 score, were used to assess and characterize the performance of the model. Supplementary Table 2 provides detailed information about the AUC values, ranging from 0.72 to 0.96, indicating good performance in most studies. Out of the total of 14 studies, 5 did not conduct any validation (Lang et al., 1997; Rau et al., 2018; Abujaber et al., 2020; Lee et al., 2022; Wang et al., 2022), 7 studies solely conducted only internal validation (Güiza et al., 2013; Matsuo et al., 2020, 2023; Pease et al., 2022; Satyadev et al., 2022; Warman et al., 2022; Song et al., 2023), while 1 study exclusively performed external validation (Tu et al., 2022). Only 1 study conducted both internal and external validation (Supplementary Table 1; Wu et al., 2023). Of the studies that performed internal validation, five used cross-validation methods (Matsuo et al., 2020; Satyadev et al., 2022; Warman et al., 2022; Song et al., 2023; Wu et al., 2023), one used bootstrap validation (Güiza et al., 2013), and the remaining two did not explicitly describe their internal validation methods (Lang et al., 1997; Wang et al., 2022). For the studies that performed external validation, one study validated the model by recruiting an additional 200 patients with similar characteristics and outcomes, while the other validated using clinical data from other centers.

### 3.2 Meta-analysis

**FIGURE 3:**
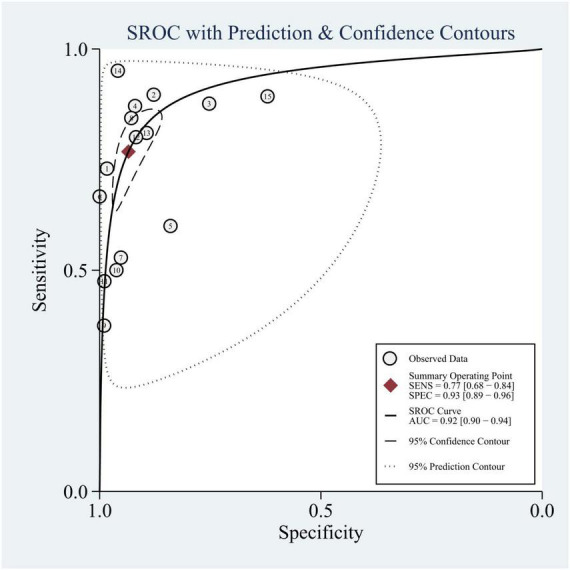
Pooled SROC curves for 14 studies.

**FIGURE 4:**
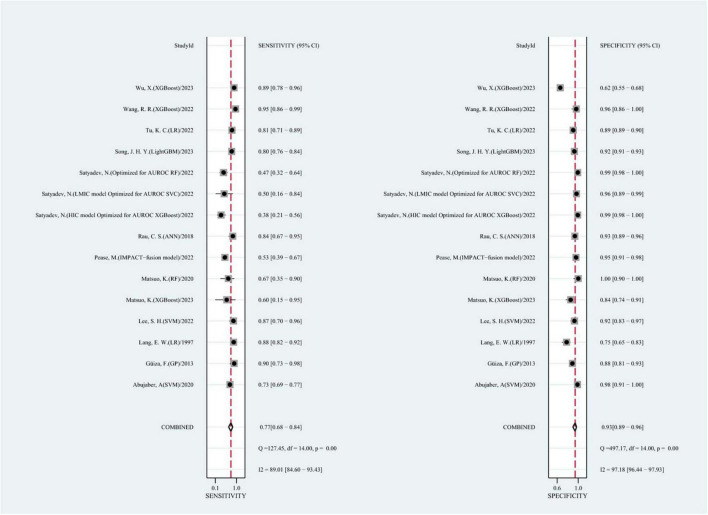
The overall pooled sensitivity and specificity of machine learning models for predicting mortality after TBI. The first author of each study was listed along the *y*-axis.

We summarized the results of 15 studies (one of which constructed two different machine learning models using two different datasets). Based on these studies, the AUC of merging was calculated as 0.90 (95% CI: 0.87 to 0.92), as shown in Figure 3. In addition, the sensitivity of merging was found to be 0.74 (95% CI: 0.69 to 0.78; *I*^2^ = 87.19%, *p* = 0.00), while the specificity of merging was determined to be 0.92 (95% CI: 0.89 to 0.94; *I*^2^ = 99.08%, *p* = 0.00), as shown in Figure 4. This data demonstrates that machine learning techniques exhibit good predictive performance for mortality in TBI patients.

**FIGURE 5:**
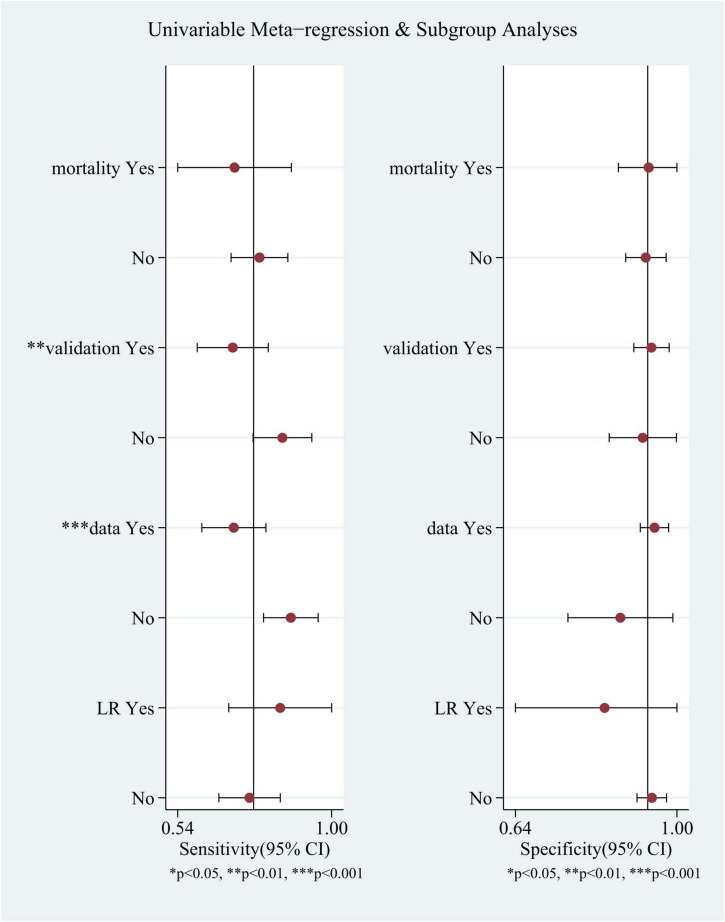
Univariable meta-regression and subgroup analyses. Comparison of sensitivity and specificity of different subgroups in TBI mortality prediction by machine learning models.

Meta-regression and subgroup analyses were performed because of the substantial heterogeneity observed in the study (Figure 5). It was speculated that this heterogeneity could be due to several factors, including whether the model was log-regressed, whether there were reports of missing data processing, whether the model was validated, and whether the outcomes were identical (such as 6-month mortality or in-hospital mortality). the same (e.g., 6-month mortality or in-hospital mortality). The results indicate that the heterogeneity in sensitivity may be attributable to the reporting of missing data handling and model validation.

### 3.3 Critical appraisal

**FIGURE 6:**
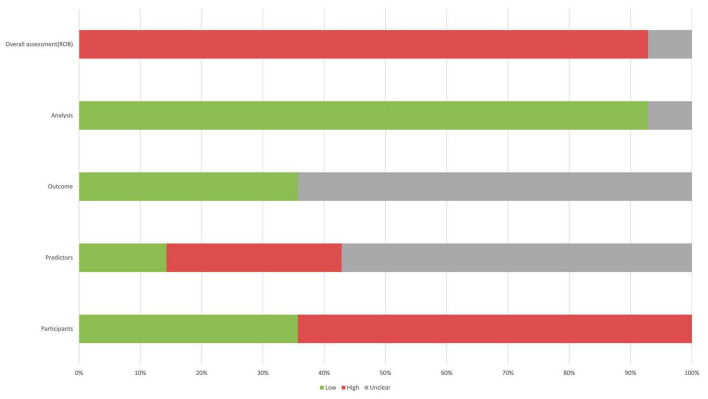
Risk of bias assessment for the predictive model studies. Study compliance with the predictive model risk of bias assessment tool (PROBAST).

**FIGURE 7:**
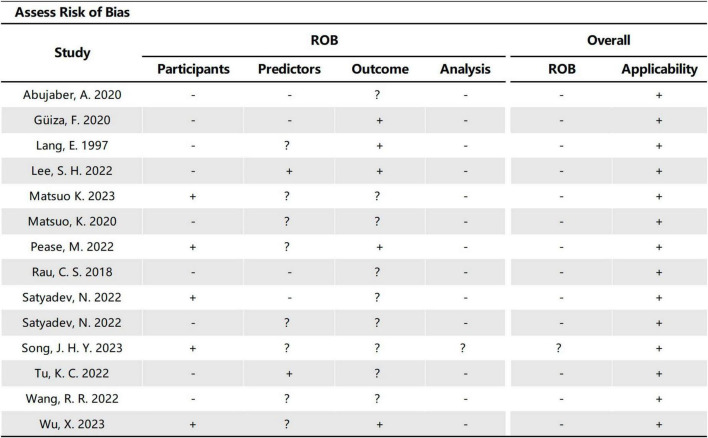
Risk of bias assessment for the included studies.

The 14 studies included in our study were assessed using PROBAST (Figures 6, 7), all considered to be at high risk of bias, with the analysis process the highest. The PROBAST tool recommends the Events Per Variable criterion (EPV) to assess overfitting. The EPV of most included studies is <10, indicating a risk of overfitting (Austin and Steyerberg, 2017). Additionally, only a few studies reported whether they considered and interpreted the complexity of the data, which is a potential reason for bias.

## 4 Discussion

**FIGURE 8:**
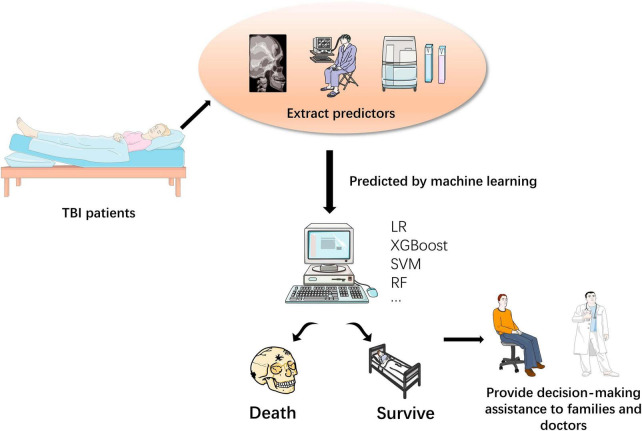
Process for predicting mortality in TBI patients using machine learning. The TBI mortality prediction model built by machine learning helps doctors and patients’ families make decisions.

Prognostic prediction of TBI has always been a critical clinical issue, especially due to the high mortality rate and potential long-term vegetative state faced by patients with moderate to severe TBI (Figure 8; Stocchetti and Zanier, 2016). Therefore, early mortality prediction plays a crucial role in helping healthcare professionals and families make informed decisions. The International Mission for Prognosis and Analysis of Clinical Trials in TBI (IMPACT) and the Corticosteroid Randomization After Significant Head Injury (CRASH) are two previously developed models that aimed to predict the prognosis of TBI patients (Bracken, 2005; MRC CRASH Trial Collaborators, Perel et al., 2008). These models utilized a sizable sample obtained from many countries and were internally and externally validated during the initial development process, demonstrating favorable performance. With the continuous advancement of machine learning technology, various machine learning algorithms have been used to build prognostic models of TBI patients, with different types of data, including data obtained from head CT scans and blood biomarkers. Comprehensive analysis of these data can effectively predict the mortality of TBI patients. However, the overall performance of these predictive models remains unclear. Therefore, this systematic review and meta-analysis aimed to assess the effectiveness of machine learning-based models in predicting mortality after TBI.

In this study, we included 15 machine learning-based predictive models from 14 studies with a total AUC = 0.90, outperforming IMPACT and CRASH in an external validation of a large dataset (Roozenbeek et al., 2012). However, the PROBAST assessment showed that these 14 studies showed a high risk of bias, which makes it challenging to accurately assess the overall performance of these predictive models. While most of the included studies were validated internally using the cross-validation methods accepted by the PROBAST tool, only one study conducted both internal and external validation, so more follow-up studies are needed to further validate the performance of the proposed model to ensure the reliability of the predictive model in clinical applications.

From data sources, all studies included case data from more than 200 people, of which eight studies involved more than 1,000 cases. However, due to the relatively limited number of events (number of deaths) in these patients, the vast majority of predictive models EPV < 10 (van Smeden et al., 2019). In addition, the quality of data for patients in retrospective studies was lower than in prospective studies, whereas most of the studies we included were retrospective. Therefore, it is better if the EPV of the included data sample is as high as possible to 20 in future studies, as recommended by the PROBAST tool, and to try to select more data from prospective studies to ensure further reliable model performance. Although the performance and reliability of the predictive models in the current research do not mean that all models using machine learning perform better and more reliably than traditional models, with the development of machine learning technology, this may indicate that the TBI predictive model based on machine learning has broader prospects in future clinical applications.

Whilst this study comprehensively explores the field of machine-learning based prediction of mortality in patients with TBI, it is important to recognize that rapid advances in machine-learning technology may lead to a significant amount of research in related areas in a short period of time. Therefore, this is one of the limitations of the current study. Furthermore, it is worth noting that this study assessed the overall performance of the included machine learning models without identifying the best performing algorithm. Consequently, further research is needed to determine the most effective algorithm. Finally, because the cohort of patients included in this study were from different countries and exhibited different medical conditions, these factors may potentially affect the predictive performance of the models.

## Data availability statement

The original contributions presented in the study are included in the article/Supplementary material, further inquiries can be directed to the corresponding authors.

## Author contributions

ZW: Writing – original draft. JL: Writing – original draft. QH: Writing – original draft. LL: Writing – review and editing. SL: Supervision, Writing – review and editing, Validation. XC: Supervision, Writing – review and editing. YH: Writing-original draft, Supervision, Writing – review and editing.
