# Supplementary material for: Machine learning-based model for predicting inpatient mortality in adults with traumatic brain injury: a systematic review and meta-analysis
Source: Front Neurosci. 2023 Dec 14;17:1285904. doi: 10.3389/fnins.2023.1285904 (PMC10753007; doi:10.3389/fnins.2023.1285904)
Supplement: Supplementary file 1 [file Table_1.DOCX]

Table 1. Characteristic of the Included Studies

| **Author，year** | **Study design** | **Country, setting** | **Sample size(n)** | **Inclusions** | **Exclusions** | **Study Outcome** | **ML algorithm** | **Missing Data** | **Internal Validation** | **External Validation** |
| --- | --- | --- | --- | --- | --- | --- | --- | --- | --- | --- |
| Abujaber, A. 2020[9] | Retrospective study | Qatar, adult patients were admitted to HGH's trauma centre between January 2014 and February 2019 and registered with the Trauma Registry | 1620 | Adult patients (≥ 14-year-old) who sustained TBI | All variables that have no predictive power (e.g. health record number, date of admission and date of disposition) or those that were severely imbalanced (e.g. gender: where female patients were less than 6%) were excluded | In-hospital mortality during the initial hospitalization post moderate to severe TBI | ANN, SVM | Records with missing data were eliminated | NR | NR |
| Güiza, F. 2013[21] | Retrospective study | 11 countries in Europe, Validated information on TBI patients in 22 neural ICUs | 264 | NR | NR | Adverse neurological outcomes such as death within 24 hours of admission or at 6 months | LR, GP | NR | "Bootstrap-corrected | NR |
| Lang, E. 1997[17] | Prospective studies | USA, between February 1977 and June 1992, patients with consecutive head injuries admitted to San Francisco General Hospital had a GCS score of 8 or less on admission or within 24 hours of hospitalization | 1204 | The data is sufficiently complete for the patient | NR | Mortality within 6 months | LR, NN | NR | NR | NR |
| Lee, S. H. 2022[10] | Retrospective study | Korea, Electronic medical records and CTs of TBI patients admitted to a single institution in Korea between February 2017 and August 2021. | 423 | NR | Patients with missing data, no abnormal CT findings, under 18 years of age, GCS score of 15, or cardiac arrest on arrival at the emergency department | Death within 14 days | LR, RF, SVM | Patients with missing data were excluded | NR | NR |
| Matsuo, K. 2023[11] | Retrospective study | Japan, Data on 200 consecutive patients treated for acute TBI at six hospitals in Hyogo Prefecture, Japan, since January 2018. | 1200 | "1) male or female participants  (> 10 years of age);  2) diagnosed with TBI requiring  hospitalization" | 1) cardiopulmonary arrest during transport or upon arrival;  2) pregnancy; 3) penetrating TBI;  4) lack of blood tests upon admission; 5) transferred after initial treatment at another institution; 6) chronic subdural hematoma;  7) injury preceded by stroke;  8) refusal to participate in the study;  9) patients with four or more missing data | At discharge in accordance with GOSE5-8; GOSE2-4; GOSE1 divided patients into three categories of outcomes | LR, XGBoost, DNN | Missing variables are filled in using k-nearest neighbor | Validate the dataset in validation | NR |
| Matsuo, K.2020[12] | Retrospective study | Japan, TBI patients admitted between October 2013 and September 2016 at Hyogo Prefectural Kakogawa Medical Center | 268 | NR | Child under 10 years old, were pregnant, or had insufficient admission laboratory or clinical  data | In-hospital poor outcome; In-hospital mortality | RF, XGBoost, ExtRa Trees, SVM, Ridge regression, LASSO regression, Gaussian NB, Multi-nomial NB, DT | Cases with missing elements were not  included | Five-fold  cross-validation | NR |
| Pease, M.2022[18] | Retrospective study | USA, patients with sTBI admitted to a level 1 trauma center from November 2002 to December 2018 | 757 | NR | Patients with pre-existing neurosurgical disease, those without an admission CT head scan prior to  neurosurgical intervention, and those who had substantial motion artifacts | Mortality and unfavorable (GOS 1–3) or favorable (GOS 4–5) outcomes at 6 months | Fusion model, Imaging model, IMPACT-fusion model | All remaining patients with incomplete data or  who lacked sufficient follow-up data were removed | Validate the dataset in validation | NR |
| Rau, C. S. 2018[13] | NR | China, inpatient adult patients enrolled in the trauma registry system of Chang Gung Hospital between 2009 and March 1734 | 2059 | NR | NR | In-hospital mortality | LR, SVM, DT, NB, ANN | Patients with missing data were not included in the dataset | NR | NR |
| (Continued) | | | | | | | | | | |
| Table 1. Continued | | | | | | | | | | |
| **Author，year** | **Study design** | **Country, setting** | **Sample size(n)** | **Inclusions** | **Exclusions** | **Study Outcome** | **ML algorithm** | **Missing Data** | **Internal Validation** | **External Validation** |
| Satyadev, N. 2022[19] | Prospective studies | Uganda, USA,  (1) National Trauma Database subset of patients from Duke University Medical Center from October 2013 to May 2020  (2) Mulago National Referral based on June 2016 to May 2020, previously created by the authors Prospective enrollment of Hospital patients | 6270 | NR | NR | In-hospital mortality | XGBoost, SVC | Missing data for any predictor variable or the outcome, including in-hospital mortality, were removed from the cohort | 5-fold cross-validation | NR |
| Satyadev, N. 2022[20] | NR | USA, patients at Duke University Hospital from October 2013 to May 2020 | 5393 | NR | NR | Mortality and dichotomized GOS at 6 months | RF | All incomplete records were subsequently removed from the data set | 5-fold cross-validation | NR |
| Song, J. H. Y. 2023[14] | Retrospective study | More than ten countries in Asia,patients (‡ 15 years) who were transported to emergency department by medical service providers between January 1, 2015, and December 31, 2020 | 3264 | NR | Patients with an unknown (GOS) score at discharge, conflicting outcomes, penetrating injury, transfer to other institutions in the ED, age <15 years, cardiopulmonary resuscitation in the EMS transport, death on arrival, or missing data on core variables | In-hospital mortality(GOS=1), fatal adverse outcomes(GOS=3) | LR, LightGBM, MLP | The missing data were imputed using multi-variate imputation with chained equations | 10-fold cross-validation | NR |
| Tu, K. C. 2022[15] | Retrospective study | China, TBI patients aged 18 years old and above admitted to the emergency room from 1 January 2010 to 31 December 2019 in the electronic medical records of three hospitals under the Chi Mei Medical Group including one medical center, one regional hospital, and one district hospital | 18249 | NR | NR | In-hospital mortality | LR, RF, SVM, LightGBM, MLP, XGBoost | NR | NR | 200 new patients with the same definitions of features and outcomes in the HIS from 10 September 2020 to 10 November 2020 for further external validation |
| Wang, R. R. 2022[16] | NR | China, patients hospitalized in West China hospital for TBI between January 2015 and June 2019 | 368 | 1) GCS on admission >13;  2) admitted to our hospital 6 hours after initial brain injury;  3) transferred from other medical centers; 4) incomplete records of included variables | NR | In-hospital mortality | LR, XGBoost | NR | NR | NR |
| Wu, X. 2023[4] | Prospective studies | Europe, China, Clinical data from sTBI patients in the CENTER-TBI China Registry and patients from the CENTER-TBI European Registry from December 2014 to December 2017 | 3917 | NR | NR | In-hospital mortality | XGBoost | Missing data were imputed with mean value. | 10-fold cross validation | CENTER-TBI European Registry were used for external validation |

ANN, Artificial neural network; SVM, Support Vector Machines; NR, not reported; LR, Logistic regression; GP, Gaussian process; NN, Neural Network; GCS, Glasgow Coma Scale; SVC, Support Vector Machines; NB, Naive Bayes; RF, Random Forest; LASSO, Least absolute shrinkage and selection operator; TBI, Traumatic brain injurie; NB, Naive Bayes; DNN, Deep Neural Networks; GOS, Glasgow Outcome Scale; XGBoost, Xtreme Gradient Boosting; Extra trees, Extremely randomized trees; CNN, Convolutional Neural Networks; DT, Decision Tree; NLP, Natural Language Processing; LightGBM, Light Gradient Boosting Machine; MLP, Multi-Layer Perceptron; IMPACT, International Mission for Prognosis and Analysis of Clinical Trials in TBI
