# Supplementary material for: Machine learning-based model for predicting inpatient mortality in adults with traumatic brain injury: a systematic review and meta-analysis
Source: Front Neurosci. 2023 Dec 14;17:1285904. doi: 10.3389/fnins.2023.1285904 (PMC10753007; doi:10.3389/fnins.2023.1285904)
Supplement: Supplementary file 2 [file Table_2.DOCX]

Table 2. Model performance

|  | | **Model performance** | | | | | |
| --- | --- | --- | --- | --- | --- | --- | --- |
| **Author, year** | **ML algorithm** | **Accuracy (%)** | **AUC (%)** | **AURPC(%)** | **Sensitivity (%)** | **Specificity (%)** | **F1 Score (%)** |
| Abujaber, A. 2020[9] | ANN | 91.6 | 93.5 | - | 62.0 | 96.0 | 64.0 |
|  | SVM | 95.6 | 96.0 | - | 73.0 | 99.0 | 80.0 |
| Güiza, F. 2013[21] | LR (IMPACT-Core Predictors) | 70.0 | 72.0 | - | 73.0 | 69.0 | - |
|  | GP (IMPACTCore + Dynamic Predictors) | 88.0 | 90.0 | - | 88.0 | 88.0 | - |
|  | GP(CRASHBasic + Dynamic Predictors) | 88.0 | 89.0 | - | 86.0 | 88.0 | - |
| Lang, E. 1997[17] | LR | 83.1 | - | - | 87.7 | 75.0 | - |
|  | NN | 85.8 | - | - | 90.6 | 77.1 | - |
| Lee, S. H. 2022[10] | LR | 88.7 | 95.0 | - | 83.9 | 90.7 | - |
|  | RF | 91.5 | 94.6 | - | 83.9 | 94.7 | - |
|  | SVM | 90.6 | 95.2 | - | 87.1 | 92.0 | - |
| Matsuo, K. 2023[11] | LR | 77.4 | 86.3 | - | 61.6 | 63.7 | - |
|  | XGBoost | 82.5 | 90.1 | - | 69.5 | 74.1 | - |
|  | DNN | 74.1 | 86.1 | - | 62.1 | 65.0 | - |
| Matsuo, K.2020[12] | RF | 89.2 | 96.0 | - | 64.4 | 99.3 | - |
|  | XGBoost | 87.6 | 95.1 | - | 73.8 | 93.2 | - |
|  | ExtRa Trees | 88.2 | 94.9 | - | 68.0 | 97.8 | - |
|  | SVM | 89.3 | 94.2 | - | 70.2 | 97.0 | - |
|  | Ridge regression | 84.9 | 93.9 | - | 85.1 | 84.9 | - |
|  | LASSO regression | 87.6 | 91.3 | - | 77.6 | 91.7 | - |
|  | Gaussian NB | 84.4 | 89.0 | - | 71.6 | 89.4 | - |
|  | Multi-nomial NB | 84.9 | 87.1 | - | 66.4 | 92.5 | - |
|  | DT | 81.1 | 81.3 | - | 68.5 | 86.3 | - |
| Pease, M.2022[18] | Fusion model | - | - | - | 66.0 | 95.0 | - |
|  | Imaging model | - | - | - | 42.0 | 95.0 | - |
|  | IMPACT-fusion model | - | - | - | 52.0 | 95.0 | - |
| Rau, C. S. 2018[13] | LR | 93.5 | - | - | 59.4 | 93.5 | - |
|  | SVM | 92.5 | - | - | 65.6 | 95.2 | - |
|  | DT | 92.9 | - | - | 43.8 | 98.3 | - |
|  | NB | 86.1 | - | - | 59.4 | 89.1 | - |
|  | ANN | 92.0 | - | - | 84.4 | 92.8 | - |
| Table 2. Continued | | | | | | | |
|  | | **Model performance** | | | | | |
| **Author, year** | **ML algorithm** | **Accuracy (%)** | **AUC (%)** | **AURPC(%)** | **Sensitivity (%)** | **Specificity (%)** | **F1 Score (%)** |
| Satyadev, N. 2022[19] | XGBoost optimized for AUROC in HIC | 97.0 | 91.0 | 53.0 | 37.0 | 99.0 | 47.0 |
|  | SVC optimized for AURPC in HIC | 97.0 | 91.0 | 50.0 | 32.0 | 99.0 | 42.0 |
|  | SVC optimized for AUROC in LMIC | 92.0 | 89.0 | 54.0 | 55.0 | 96.0 | 55.0 |
|  | SVC optimized for AURPC in LMIC | 89.0 | 90.0 | 52.0 | 52.0 | 93.0 | 48.0 |
| Satyadev, N. 2022[19] | RF optimized for AUROC | 83.0 | 84.0 | 85.0 | 81.0 | 50.0 | 79.0 |
|  | RF optimized for AURPC | 83.0 | 84.0 | 85.0 | 81.0 | 50.0 | 79.0 |
| Song, J. H. Y. 2023[14] | LR | - | - | - | 80.0 | 87.5 | - |
|  | LightGBM | - | - | - | 80.0 | 91.8 | - |
|  | MLP | - | - | - | 80.0 | 88.5 | - |
| Tu, K. C. 2022[15] | LR | 89.3 | 92.5 | - | 81.2 | 89.4 | - |
|  | RF | 80.0 | 87.0 | - | 80.0 | 80.0 | - |
|  | SVM | 86.5 | 92.0 | - | 86.2 | 86.5 | - |
|  | LightGBM | 70.8 | 85.1 | - | 82.5 | 70.6 | - |
|  | MLP | 82.5 | 89.3 | - | 82.5 | 82.5 | - |
|  | XGBoost | 71.7 | 87.1 | - | 83.8 | 71.5 | - |
| Wang, R. R. 2022[16] | LR | 70.3 | 80.5 | - | 73.8 | 75.4 | - |
|  | XGBoost | 95.5 | 95.5 | - | 94.5 | 96.4 | - |
| Wu, X. 2023[4] | XGBoost | - | - | - | 64.0 | 90.0 | - |

ANN, Artificial Neural Network; SVM, Support Vector Machines; LR, Logistic regression; GP, Gaussian Process; NN, Neural Network; SVC, Support Vector Machines; NB, Naive Bayes; RF, Random Forest; LASSO, Least absolute shrinkage and selection operator; NB, Naive Bayes; DNN, Deep Neural Networks; XGBoost, Xtreme Gradient Boosting; Extra trees, Extremely randomized trees; DT, Decision Tree; LightGBM, Light Gradient Boosting Machine; MLP, Multi-Layer Perceptron; IMPACT, International Mission for Prognosis and Analysis of Clinical Trials in TBI
