# Supplementary material for: Machine learning-based model for predicting inpatient mortality in adults with traumatic brain injury: a systematic review and meta-analysis
Source: Front Neurosci. 2023 Dec 14;17:1285904. doi: 10.3389/fnins.2023.1285904 (PMC10753007; doi:10.3389/fnins.2023.1285904)
Supplement: Supplementary file 3 [file Data_Sheet_1.docx]

Literature search strategy

**1.Pubmed**

| Search number | Query | Results |
| --- | --- | --- |
| #1 | ((((((((((((((("Brain Injuries, Traumatic"[Mesh]) OR (Brain Injury, Traumatic[Title/Abstract])) OR (Traumatic Brain Injuries[Title/Abstract])) OR (Trauma, Brain[Title/Abstract])) OR (Brain Trauma[Title/Abstract])) OR (Brain Traumas[Title/Abstract])) OR (Traumas, Brain[Title/Abstract])) OR (TBI (Traumatic Brain Injury[Title/Abstract]))) OR (Encephalopathy, Traumatic[Title/Abstract])) OR (Encephalopathies, Traumatic[Title/Abstract])) OR (Traumatic Encephalopathies[Title/Abstract])) OR (Injury, Brain, Traumatic[Title/Abstract])) OR (Traumatic Encephalopathy[Title/Abstract])) OR (TBIs (Traumatic Brain Injuries[Title/Abstract]))) OR (TBI (Traumatic Brain Injuries[Title/Abstract]))) OR (Traumatic Brain Injury[Title/Abstract]) |  |
| #2 | ((((((((((((((((((((((((((((((((((((((("Mortality"[Mesh]) OR (Mortalities[Title/Abstract])) OR (Case Fatality Rate[Title/Abstract])) OR (Case Fatality Rates[Title/Abstract])) OR (Rate, Case Fatality[Title/Abstract])) OR (Rates, Case Fatality[Title/Abstract])) OR (CFR Case Fatality Rate[Title/Abstract])) OR (Crude Death Rate[Title/Abstract])) OR (Crude Death Rates[Title/Abstract])) OR (Death Rate, Crude[Title/Abstract])) OR (Rate, Crude Death[Title/Abstract])) OR (Crude Mortality Rate[Title/Abstract])) OR (Crude Mortality Rates[Title/Abstract])) OR (Mortality Rate, Crude[Title/Abstract])) OR (Rate, Crude Mortality[Title/Abstract])) OR (Death Rate[Title/Abstract])) OR (Death Rates[Title/Abstract])) OR (Rate, Death[Title/Abstract])) OR (Mortality Rate[Title/Abstract])) OR (Mortality Rates[Title/Abstract])) OR (Rate, Mortality[Title/Abstract])) OR (Mortality, Excess[Title/Abstract])) OR (Excess Mortality[Title/Abstract])) OR (Excess Mortalities[Title/Abstract])) OR (Decline, Mortality[Title/Abstract])) OR (Mortality Declines[Title/Abstract])) OR (Mortality Decline[Title/Abstract])) OR (Mortality Determinants[Title/Abstract])) OR (Determinants, Mortality[Title/Abstract])) OR (Determinant, Mortality[Title/Abstract])) OR (Mortality Determinant[Title/Abstract])) OR (Mortality, Differential[Title/Abstract])) OR (Differential Mortality[Title/Abstract])) OR (Differential Mortalities[Title/Abstract])) OR (Age-Specific Death Rate[Title/Abstract])) OR (Age-Specific Death Rates[Title/Abstract])) OR (Death Rate, Age-Specific[Title/Abstract])) OR (Rate, Age-Specific Death[Title/Abstract])) OR (Age Specific Death Rate[Title/Abstract])) OR (((((("Risk"[Mesh]) OR (risk[Title/Abstract])) OR (Relative Risk[Title/Abstract])) OR (Relative Risks[Title/Abstract])) OR (Risk, Relative[Title/Abstract])) OR (Risks, Relative[Title/Abstract])) |  |
| #3 | ((((((((("Machine Learning"[Mesh]) OR (Learning, Machine[Title/Abstract])) OR (Transfer Learning[Title/Abstract])) OR (Learning, Transfer[Title/Abstract])) OR (((((((((((((((((((("Artificial Intelligence"[Mesh]) OR (Intelligence, Artificial[Title/Abstract])) OR (Computational Intelligence[Title/Abstract])) OR (Intelligence, Computational[Title/Abstract])) OR (Machine Intelligence[Title/Abstract])) OR (Intelligence, Machine[Title/Abstract])) OR (Computer Reasoning[Title/Abstract])) OR (Reasoning, Computer[Title/Abstract])) OR (AI (Artificial Intelligence[Title/Abstract]))) OR (Computer Vision Systems[Title/Abstract])) OR (Computer Vision System[Title/Abstract])) OR (System, Computer Vision[Title/Abstract])) OR (Systems, Computer Vision[Title/Abstract])) OR (Vision System, Computer[Title/Abstract])) OR (Vision Systems, Computer[Title/Abstract])) OR (Knowledge Acquisition (Computer[Title/Abstract]))) OR (Acquisition, Knowledge (Computer[Title/Abstract]))) OR (Knowledge Representation (Computer[Title/Abstract]))) OR (Knowledge Representations (Computer[Title/Abstract]))) OR (Representation, Knowledge (Computer[Title/Abstract])))) OR (((("Deep Learning"[Mesh]) OR (Learning, Deep[Title/Abstract])) OR (Hierarchical Learning[Title/Abstract])) OR (Learning, Hierarchical[Title/Abstract]))) OR (((((((((((((((((((((((((((("Neural Networks, Computer"[Mesh]) OR (Computer Neural Network[Title/Abstract])) OR (Computer Neural Networks[Title/Abstract])) OR (Network, Computer Neural[Title/Abstract])) OR (Networks, Computer Neural[Title/Abstract])) OR (Neural Network, Computer[Title/Abstract])) OR (Models, Neural Network[Title/Abstract])) OR (Model, Neural Network[Title/Abstract])) OR (Network Model, Neural[Title/Abstract])) OR (Network Models, Neural[Title/Abstract])) OR (Neural Network Model[Title/Abstract])) OR (Neural Network Models[Title/Abstract])) OR (Computational Neural Networks[Title/Abstract])) OR (Computational Neural Network[Title/Abstract])) OR (Network, Computational Neural[Title/Abstract])) OR (Networks, Computational Neural[Title/Abstract])) OR (Neural Network, Computational[Title/Abstract])) OR (Neural Networks, Computational[Title/Abstract])) OR (Perceptrons[Title/Abstract])) OR (Perceptron[Title/Abstract])) OR (Connectionist Models[Title/Abstract])) OR (Connectionist Model[Title/Abstract])) OR (Model, Connectionist[Title/Abstract])) OR (Models, Connectionist[Title/Abstract])) OR (Neural Networks (Computer[Title/Abstract]))) OR (Network, Neural (Computer[Title/Abstract]))) OR (Networks, Neural (Computer[Title/Abstract]))) OR (Neural Network (Computer[Title/Abstract])))) OR ((((((((Random Forests[Title/Abstract]) OR ("Random Forest"[Mesh])) OR (Random Forest Classification[Title/Abstract])) OR (Classification, Random Forest[Title/Abstract])) OR (Random Forest Classifications[Title/Abstract])) OR (Random Forest Algorithm[Title/Abstract])) OR (Algorithm, Random Forest[Title/Abstract])) OR (Random Forest Algorithms[Title/Abstract]))) OR (((("Decision Trees"[Mesh]) OR (Decision Tree[Title/Abstract])) OR (Tree, Decision[Title/Abstract])) OR (Trees, Decision[Title/Abstract]))) OR (((((((((((("Support Vector Machine"[Mesh]) OR (Machine, Support Vector[Title/Abstract])) OR (Machines, Support Vector[Title/Abstract])) OR (Support Vector Machines[Title/Abstract])) OR (Vector Machine, Support[Title/Abstract])) OR (Vector Machines, Support[Title/Abstract])) OR (Support Vector Network[Title/Abstract])) OR (Network, Support Vector[Title/Abstract])) OR (Networks, Support Vector[Title/Abstract])) OR (Support Vector Networks[Title/Abstract])) OR (Vector Network, Support[Title/Abstract])) OR (Vector Networks, Support[Title/Abstract])) |  |
| #4 | #1 AND #2 AND #3 | 112 |

**2.Embase**

| Search number | Query | Results |
| --- | --- | --- |
| #1 | ‘Brain Injury, Traumatic’:ab,kw,ti OR ‘Traumatic Brain Injuries’:ab,kw,ti OR ‘Trauma, Brain’:ab,kw,ti OR ‘Brain Trauma’:ab,kw,ti OR ‘Brain Traumas’:ab,kw,ti OR ‘Traumas, Brain’:ab,kw,ti OR ‘TBI Traumatic Brain Injury’:ab,kw,ti OR ‘Encephalopathy, Traumatic’:ab,kw,ti OR ‘Encephalopathies, Traumatic’:ab,kw,ti OR ‘Traumatic Encephalopathies’:ab,kw,ti OR ‘ Injury, Brain, Traumatic’:ab,kw,ti OR ‘Traumatic Encephalopathy’:ab,kw,ti OR ‘TBIs Traumatic Brain Injuries’:ab,kw,ti OR ‘TBI Traumatic Brain Injuries’:ab,kw,ti OR ‘Traumatic Brain Injury':ab,kw,ti |  |
| #2 | ‘Brain Injury, Traumatic’:ab,kw,ti OR ‘Traumatic Brain Injuries’:ab,kw,ti OR ‘Trauma, Brain’:ab,kw,ti OR ‘Brain Trauma’:ab,kw,ti OR ‘Brain Traumas’:ab,kw,ti OR ‘Traumas, Brain’:ab,kw,ti OR ‘TBI Traumatic Brain Injury’:ab,kw,ti OR ‘Encephalopathy, Traumatic’:ab,kw,ti OR ‘Encephalopathies, Traumatic’:ab,kw,ti OR ‘Traumatic Encephalopathies’:ab,kw,ti OR ‘ Injury, Brain, Traumatic’:ab,kw,ti OR ‘Traumatic Encephalopathy’:ab,kw,ti OR ‘TBIs Traumatic Brain Injuries’:ab,kw,ti OR ‘TBI Traumatic Brain Injuries’:ab,kw,ti OR ‘Traumatic Brain Injury':ab,kw,ti |  |
| #3 | ‘Machine Learning’:ab,kw,ti OR ‘Learning, Machine’:ab,kw,ti OR ‘Transfer Learning’:ab,kw,ti OR ‘Learning, Transfer’:ab,kw,ti OR ‘Artificial Intelligence’:ab,kw,ti OR ‘Intelligence, Artificial’:ab,kw,ti OR ‘Computational Intelligence’:ab,kw,ti OR ‘Intelligence, Computational’:ab,kw,ti OR ‘Machine Intelligence’:ab,kw,ti OR ‘Intelligence, Machine’:ab,kw,ti OR ‘Computer Reasoning’:ab,kw,ti OR ‘Reasoning, Computer’:ab,kw,ti OR ‘AI Artificial Intelligence’:ab,kw,ti OR ‘Computer Vision Systems’:ab,kw,ti OR ‘Computer Vision System’:ab,kw,ti OR ‘System, Computer Vision’:ab,kw,ti OR ‘Systems, Computer Vision’:ab,kw,ti OR ‘Vision System, Computer’:ab,kw,ti OR ‘Vision Systems, Computer’:ab,kw,ti OR ‘Knowledge Acquisition Computer’:ab,kw,ti OR ‘Acquisition, Knowledge Computer’:ab,kw,ti OR ‘Knowledge Representation Computer’:ab,kw,ti OR ‘Knowledge Representations Computer’:ab,kw,ti OR ‘Representation, Knowledge Computer’:ab,kw,ti OR ‘Deep Learning’:ab,kw,ti OR ‘Learning, Deep’:ab,kw,ti OR ‘Hierarchical Learning’:ab,kw,ti OR ‘Learning, Hierarchical’:ab,kw,ti OR ‘Neural Networks, Computer’:ab,kw,ti OR ‘Computer Neural Network’:ab,kw,ti OR ‘Computer Neural Networks’:ab,kw,ti OR ‘Network, Computer Neural’:ab,kw,ti OR ‘Networks, Computer Neural’:ab,kw,ti OR ‘Neural Network, Computer’:ab,kw,ti OR ‘Models, Neural Network’:ab,kw,ti OR ‘Model, Neural Network’:ab,kw,ti OR ‘Network Model, Neural’:ab,kw,ti OR ‘Network Models, Neural’:ab,kw,ti OR ‘Neural Network Model’:ab,kw,ti OR ‘Neural Network Models’:ab,kw,ti OR ‘Computational Neural Networks’:ab,kw,ti OR ‘Computational Neural Network’:ab,kw,ti OR ‘Network, Computational Neural’:ab,kw,ti OR ‘Networks, Computational Neural’:ab,kw,ti OR ‘Neural Network, Computational’:ab,kw,ti OR ‘Neural Networks, Computational’:ab,kw,ti OR ‘Perceptrons’:ab,kw,ti OR ‘Perceptron’:ab,kw,ti OR ‘Connectionist Models’:ab,kw,ti OR ‘Connectionist Model’:ab,kw,ti OR ‘Model, Connectionist’:ab,kw,ti OR ‘Models, Connectionist’:ab,kw,ti OR ‘Neural Networks Computer’:ab,kw,ti OR ‘Network, Neural Computer’:ab,kw,ti OR ‘Networks, Neural Computer’:ab,kw,ti OR ‘Neural Network Computer’:ab,kw,ti OR ‘Random Forests’:ab,kw,ti OR ‘Random Forest’:ab,kw,ti ‘OR ‘Random Forest Classification’:ab,kw,ti OR ‘Classification, Random Forest’:ab,kw,ti OR ‘Random Forest Classifications’:ab,kw,ti OR ‘Random Forest Algorithm’:ab,kw,ti OR ‘Algorithm, Random Forest’:ab,kw,ti OR ‘Random Forest Algorithms’:ab,kw,ti OR ‘Decision Trees’:ab,kw,ti OR ‘Decision Tree’:ab,kw,ti OR ‘Tree, Decision’:ab,kw,ti OR ‘Trees, Decision’:ab,kw,ti OR ‘Support Vector Machine’:ab,kw,ti OR ‘Machine, Support Vector’:ab,kw,ti OR ‘Machines, Support Vector’:ab,kw,ti OR ‘Support Vector Machines’:ab,kw,ti OR ‘Vector Machine, Support’:ab,kw,ti OR ‘Vector Machines, Support’:ab,kw,ti OR ‘Support Vector Network’:ab,kw,ti OR ‘Network, Support Vector’:ab,kw,ti OR ‘Networks, Support Vector’:ab,kw,ti OR ‘Support Vector Networks’:ab,kw,ti OR ‘Vector Network, Support’:ab,kw,ti OR ‘Vector Networks, Support’:ab,kw,ti |  |
| #4 | #1 AND #2 AND #3 | 198 |

**3.Web of science**

| Search number | Query | Results |
| --- | --- | --- |
| #1 | Brain Injuries, Traumatic (Topic) or Brain Injury, Traumatic (Topic) or Traumatic Brain Injuries (Topic) or Trauma, Brain (Topic) or Brain Trauma (Topic) or Brain Traumas (Topic) or Traumas, Brain (Topic) or TBI (Traumatic Brain Injury) (Topic) or Encephalopathy, Traumatic (Topic) or Encephalopathies, Traumatic (Topic) or Traumatic Encephalopathies (Topic) or Injury, Brain, Traumatic (Topic) or Injury, Brain, Traumatic (Topic) or TBIs (Traumatic Brain Injuries) (Topic) or TBI (Traumatic Brain Injuries) (Topic) or Traumatic Brain Injury (Topic |  |
| #2 | Risk (Topic) or Risks (Topic) or Relative Risk (Topic) or Relative Risks (Topic) or Risk, Relative (Topic) or Risks, Relative (Topic) or Mortality (Topic) or Mortalities (Topic) or Case Fatality Rate (Topic) or Case Fatality Rates (Topic) or Rate, Case Fatality (Topic) or Rates, Case Fatality (Topic) or CFR Case Fatality Rate (Topic) or Crude Death Rate (Topic) or Crude Death Rates (Topic) or Death Rate, Crude (Topic) or Rate, Crude Death (Topic) or Crude Mortality Rate (Topic) or Crude Mortality Rates (Topic) or Mortality Rate, Crude (Topic) or Rate, Crude Mortality (Topic) or Death Rate (Topic) or Death Rates (Topic) or Rate, Death (Topic) or Mortality Rate (Topic) or Mortality Rates (Topic) or Rate, Mortality (Topic) or Mortality, Excess (Topic) or Excess Mortality (Topic) or Excess Mortalities (Topic) or Decline, Mortality (Topic) or Mortality Declines (Topic) or Mortality Decline (Topic) or Mortality Determinants (Topic) or Determinants, Mortality (Topic) or Determinant, Mortality (Topic) or Mortality Determinant (Topic) or Mortality, Differential (Topic) or Differential Mortality (Topic) or Differential Mortalities (Topic) or Age-Specific Death Rate (Topic) or Age-Specific Death Rates (Topic) or Death Rate, Age-Specific (Topic) or Rate, Age-Specific Death (Topic) or Age Specific Death Rate (Topic) |  |
| #3 | (Topic) or Support Vector Network (Topic) or Network, Support Vector (Topic) or Networks, Support Vector (Topic) or Support Vector Networks (Topic) or Vector Network, Support (Topic) or Vector Networks, Support (Topic) Machine Learning (Topic) or Learning, Machine (Topic) or Transfer Learning (Topic) or Learning, Transfer (Topic) or Artificial Intelligence (Topic) or Intelligence, Artificial (Topic) or Computational Intelligence (Topic) or Intelligence, Computational (Topic) or Machine Intelligence (Topic) or Intelligence, Machine (Topic) or Computer Reasoning (Topic) or Reasoning, Computer (Topic) or AI (Artificial Intelligence) (Topic) or Computer Vision Systems (Topic) or Computer Vision System (Topic) or System, Computer Vision (Topic) or Systems, Computer Vision (Topic) or Vision System, Computer (Topic) or Vision Systems, Computer (Topic) or Knowledge Acquisition (Computer) (Topic) or Acquisition, Knowledge (Computer) (Topic) or Knowledge Representation (Computer) (Topic) or Knowledge Representations (Computer) (Topic) or Representation, Knowledge (Computer) (Topic) or Deep Learning (Topic) or Learning, Deep (Topic) or Hierarchical Learning (Topic) or Learning, Hierarchical (Topic) or Neural Networks, Computer (Topic) or Computer Neural Network (Topic) or Computer Neural Networks (Topic) or Network, Computer Neural (Topic) or Networks, Computer Neural (Topic) or Neural Network, Computer (Topic) or Models, Neural Network (Topic) or Model, Neural Network (Topic) or Network Model, Neural (Topic) or Network Models, Neural (Topic) or Neural Network Model (Topic) or Neural Network Models (Topic) or Computational Neural Networks (Topic) or Computational Neural Network (Topic) or Network, Computational Neural (Topic) or Networks, Computational Neural (Topic) or Neural Network, Computational (Topic) or Neural Networks, Computational (Topic) or Perceptrons (Topic) or Perceptron (Topic) or Connectionist Models (Topic) or Connectionist Model (Topic) or Model, Connectionist (Topic) or Models, Connectionist (Topic) or Neural Networks (Computer) (Topic) or Network, Neural (Computer) (Topic) or Networks, Neural (Computer) (Topic) or Neural Network (Computer) (Topic) or Random Forest (Topic) or Random Forests (Topic) or Random Forest Classification (Topic) or Classification, Random Forest (Topic) or Random Forest Classifications (Topic) or Random Forest Algorithm (Topic) or Algorithm, Random Forest (Topic) or Random Forest Algorithms (Topic) or Decision Trees (Topic) or Decision Tree (Topic) or Tree, Decision (Topic) or Trees, Decision (Topic) or Support Vector Machine (Topic) or Machine, Support Vector (Topic) or Machines, Support Vector (Topic) or Support Vector Machines (Topic) or Vector Machine, Support (Topic) or Vector Machines, Support |  |
| #4 | #1 AND #2 AND #3 | 308 |
